# Evaluation of the relationship between body composition and dietary habits of physically active people with disabilities

**DOI:** 10.1038/s41598-024-60735-x

**Published:** 2024-05-04

**Authors:** Agnieszka Pelc, Katarzyna Walicka-Cupryś, Gabriela Puszkarz, Kamila Styś, Elżbieta Chmiel, Sebastian Wilk, Grzegorz Ludwikowski, Katarzyna Placek

**Affiliations:** 1 Student Scientific Circle-Fascination With Body Posture “Habitus”, University of Rzeszów, Rejtana 16C Street, 35-959 Rzeszow, Poland; 2 Faculty of Health Sciences, College of Medical Sciences, University of Rzeszów, Rejtana 16C Street, 35-959 Rzeszow, Poland; 3 Department and Clinic of Obstetrics, Women’s Diseases and Oncological Gynecology, Collegium Medicum in Bydgoszcz, Nicolaus Copernicus University, University Hospital No. 2 Jana Biziela in Bydgoszcz, 85-067 Bydgoszcz, Poland

**Keywords:** Health care, Health occupations, Medical research

## Abstract

Physical activity offers numerous physical and mental health benefits for individuals with disabilities, while nutrition plays a crucial role in maintaining bodily homeostasis. This study aimed to assess the relationship between body composition and dietary habits among physically active people with disabilities. Fifty-five participants aged 16 to 61, including 28 with disabilities and 27 without, were included in the study. The FFQ-6 questionnaire, Tanita body composition analyzer, and Stadiometer were utilized. No significant differences in BMI were observed between the two groups. However, individuals with disabilities showed higher body fat, metabolic age, or pulse values, whereas the control group exhibited higher muscle mass, muscle quality, body type, or bone mass. Participants with disabilities were more likely to consume vegetables (p = 0.004) and animal fats (p = 0.027), while those without disabilities were more inclined to consume fast food, instant products (p = 0.006), sweetened beverages (p < 0.001), and alcohol (p < 0.001). People with disabilities often have a higher percentage of body fat, cautioning against the consumption of processed fruits, dried fruits, fast food, and red meat. Conversely, in non-disabled individuals, frequent consumption of eggs, animal fats, sugar, and sweets is not recommended due to the potential for increased body fat, visceral fat, and higher BMI.

## Introduction

Physical activity provides opportunities for people with disabilities to experience a range of physical and mental health benefits^1^. These benefits include improved function, strength and muscle tone, increased opportunities for socialisation and reduced anxiety and depression^2^. Furthermore, physically inactive people with disabilities also have higher rates of chronic diseases, i.e. cancer, diabetes, heart disease, stroke^3^. Sport for people with disabilities serves not only a therapeutic function, but has also an anatomical-physiological, educational-psychological, integrative and compensatory function^4^.

According to the new recommendations, adults aged 18–64 years should do 150–300 min of moderate-intensity or 75–150 min of high-intensity physical activity per week, while among children and adolescents, an average of 60 min per day of moderate- or high-intensity aerobic activity is required^5^. Activity levels among adults with disabilities have increased in recent years. The number of people who performed physical activity ≥ 150 min/week increased from 43.6 to 47.3%, and ˂ 30 min/week, decreased from 42.4 to 39.8%^6^.

Nutrition is one of the main determinants of the body's homeostasis, activities such as the preparation of meals, the way in which meals are acquired, stored, composed, the frequency and quantity of consumption of given foods belong to the health behaviours^7^. With proper nutrition, life can be significantly prolonged and, conversely, when nutrition is inappropriate, it can reduce life expectancy. Currently, the trend of 'overfeeding and undernutrition' is noted, meaning, an excess of calories consumed and a lack of essential nutrients, which in turn leads to deficiencies, excess chronic diseases, excessive body weight and increased mortality rates^8^. Therefore, body mass composition as an indicator of health and nutritional status is part of maintaining good health and well-being. One tool for assessing body mass composition is BIA—bioelectrical impedance analysis. The BIA test uses a low-intensity electric current; this current flows through the subject's body or encounters resistance during the test. The electric current flows easily through tissues that are well hydrated due to their high electrolyte content, while adipose tissue constitutes resistance to the flow of current^9^. BIA investigates the relationship between cellular water status and cell membrane integrity and cell mass. Bioimpedance testing is based on the principle of Ohm's law, which proves that the potential difference or voltage is directly related to the resistance to current flow^10^.

While there are studies on the effect of frequency of consumption of given foods on body weight composition, there are no studies focusing on the analysis of body composition and eating habits of physically active people with disabilities. Our study adds to the information on body composition of physically active people with disabilities compared to physically active people without disabilities. Consequently, it can provide valuable insights into how to improve their health, fitness and quality of life. The aim of this study is to assess body mass composition and eating patterns and habits in relation to disability in a group of physically active people.

## Material and methods

### Study participants

The study included fifty-five physically active people aged 16–61 years, with an average of 31.5 ± 10.89. There were 28 people in the study group and 27 people in the control group. The study group consisted of people with disabilities (i.e., musculoskeletal diseases and developmental and mental disorders) who regularly trained Frame Running. The control group consisted of non-disabled people matched to the study group in terms of age, gender and amount of physical activity, training in aerobic activity in fitness clubs in the Podkarpackie region of Poland. There were slightly more men than women in both groups. There were a total of 31 men, 56.4%, and 24 women, 43.6%. There were no differences between the gender of the subjects in the two groups (p = 0.906). The subjects in the two groups differed in body height (p = 0.001), the subjects in the control group were taller. There were no differences between the subjects' ages, or in terms of their weight and BMI (p > 0.05). The exact characteristics of the anthropometric features according to the group are shown in Table 1.

**Table 1:** Anthropometric measurements.

	Study group					Control group					t	p
	X	Me	Min	Max	S	X	Me	Min	Max	S		
Age	31.5	29.5	16.0	61.0	10.9	31.7	29.0	17.0	61.0	10.9	− 0.07	0.945
Body height	165.4	166.0	149.0	183.0	9.8	174.0	175.0	151.0	189.0	8.1	**− 3.57**	**0.001**
Body weight	70.2	68.7	40.0	108.9	15.0	73.0	73.1	45.0	110.3	13.9	− 0.73	0.468
BMI	25.9	25.1	15.4	36.8	5.2	24.2	23.0	17.6	33.2	3.3	1.47	0.146

X: arithmetic mean; Me: median; Min: minimum; Max: maximum; S: sample standard deviation; Student's t: test value for independent variables; p: test probability ratio.

Significant values are in bold.

### Study qualification

Inclusion criteria for the study group were: regular Frame Running training 1 time per week 2 h, age 16–61 years, diagnosed musculoskeletal disease, developmental and mental disorders, informed consent to participate in the study. Inclusion criteria for the control group were: regular physical activity 2 h per week, age 16–61 years, non-disabled, informed consent to participate in the study. Exclusion criteria for the study group were as follows: non-trainer or non-regular Frame Running trainer, age < 16 and > 61 years, non-disabled, physically inactive, no informed consent to participate in the study. Exclusion criteria for the control group were as follows: physically inactive subjects, age < 16 and > 61 years, diagnosed musculoskeletal disease, developmental and mental disorders, no consent to participate in the study.

**Figure 1:**
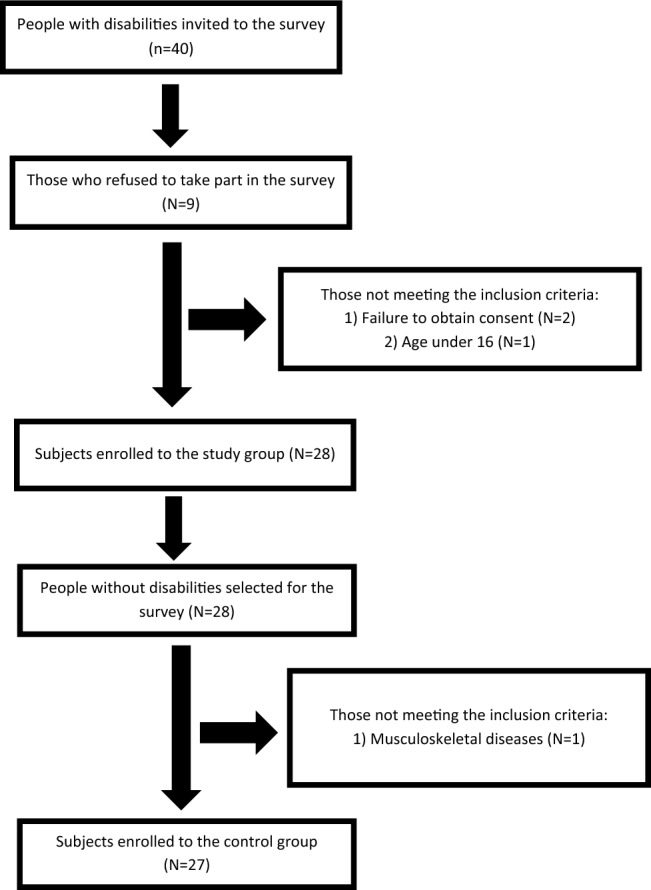
Flow chart of the study participants.

The study consisted of six stages. In the first stage, invitations (N = 40) to participate in the study were sent to people with disabilities who regularly trained in Frame Running once a week for 2 h. Frame Running is an adaptive para-athletic discipline in which one exercises on a tricycle frame without pedals, with a saddle and front chest support^11^. The second stage involved 28 participants aged 16–61 years. This stage included an anthropometric study of height and body mass composition. In the third stage, participants completed an anonymous nutrition questionnaire. In the fourth stage, non-disabled participants were matched for age, gender and amount of physical activity. These were individuals who trained in a fitness club with a personal trainer for 2 h per week. Twenty-eight participants from the control group progressed to the fifth stage, in which an anthropometric study of height and body mass composition was conducted. In the final stage, participants completed an anonymous nutrition questionnaire. After taking into account the exclusion criteria, 27 participants were included in the control group. The exact flow of the subjects is shown in Fig. 1.

The research project was approved by the institutional Bioethics Committee at the University of Rzeszow (Resolution No. 5/112014) and was carried out in accordance with the Helsinki Declaration. Each person participating in it signed voluntarily and consciously consent to participate in the study. Informed consent was obtained from all participants to participate in the study without compensation.

### Methods

#### Anthropometric and body composition measurements

Participants entered the study fasting and abstained from exercise, alcohol and stimulant drinks for at least 15 h. All the measurements were performed on the same day, starting with the anthropometric measurements^12^. The measurements were performed under standard conditions; participants in underwear and barefoot stood upright without bending the knees. Body height was measured with a Seca 213 mobile stadiometer, with an accuracy of 0.1 cm.

Electrical bioimpedance testing was carried out with a Tanita RD 545 HR body composition analyser. Body mass composition was examined, including components: weight; BMI: body mass index; BF (%): body fat; MM (kg): muscle mass; MQ (mq): muscle quality; BT: body type; BM (kg): bone mass; VF: visceral fat; PMR: primary metabolic rate; MA: metabolic age; BWC (%): body water content; BFC (%): body fat corpus; LAF (%): left arm fat; RAF (%): right arm fat; MMC (kg): muscle mass corpus; MMLA (kg): muscle mass left arm; MMRA (kg): muscle mass right arm; MMLL (kg): muscle mass left leg; MMRL (kg): muscle mass right leg; MQLA (mq): muscle quality left arm; MQRA (mq): muscle quality right arm; MQLL (mq): muscle quality left leg; MQRL (mq): muscle quality right leg.

#### Questionnaire

The basic questions concerned such data as age, place of residence, education and the possible presence of diseases and/or disabilities. The main part of the questionnaire included questions on the frequency of consumption of the types of food in question. The FFQ-6 questionnaire was used for this purpose, and the consumption of 62 food groups over the past 12 months was assessed using this scale. When answering to indicate the frequency of consumption, respondents could choose one of six categories: 1: never or almost never; 2: once a month or less often; 3: several times a month; 4: several times a week 5: every day; 6: several times a day^13^. The 62 food groups were then divided into 16 categories. The exact division of these foods is shown in Table 2.

**Table 2:** Categorisation of food products.

Category	Product groups
Sugar and sweets	Chocolates, candy bars, waffles Non-chocolate candies, e.g. jelly beans, fudge Biscuits and cakes, e.g. shortcakes, cheesecakes, doughnuts, yeast cakes Ice cream, pudding
Salty snacks	Crisps, sticks, crackers
Milk and milk products	cheese, French cheese, processed cheese, spreadable cheese, feta Cottage cheese, natural cottage cheese, cottage cheese, mozzarella Natural yoghurts, natural skyrs, sugar-free yoghurts Sweetened yoghurts, e.g. fruit yoghurts, yoghurts with flakes, fruit buttermilk, flavoured cottage cheese Protein yoghurts, protein puddings, protein cheeses, fit yoghurts, protein drinking yoghurts Milk, kefir, buttermilk
Eggs	Eggs, egg dishes, omelettes, boiled eggs
Red meat and meat products	Pork, beef, veal Game (wild boar, roe deer, wild duck meat) Sausage Ham Sausages, pâtés, black pudding, brawn Kabanos Bacon
White meat and fish	Poultry meat: chicken, duck, turkey Fatty fish, e.g. salmon, sardines, herring, mackerel, carp, eel Lean fish, e.g. pollock, cod, perch, tuna, panga, trout
Vegetables	Vegetables (all types) Cruciferous vegetables: white cabbage, savoy cabbage, red cabbage, Brussels sprouts, cauliflower, kale, broccoli Yellow-orange vegetables: peppers, carrots Green vegetables: spinach, lettuce, rocket, celery Tomatoes Cucumbers, squash, courgettes, pumpkins, aubergines Root vegetables: beetroot, parsley, garlic, onion, radish, turnip Maize, green peas, beans Broad beans, beans, soya beans, peas, chickpeas Boiled and baked potatoes Fries, potato pancakes Soups, cream soups
Nuts, grains	Nuts, e.g. peanuts, hazelnuts, walnuts, almonds, pistachios, nut creams, e.g. peanut butter Seeds, e.g. pumpkin, sesame, sunflower
Fruit	Fruit (all types) Stone fruits: apricots, avocados, cherries, nectarines, plums Kiwi and citrus: orange, madarine, lemon, pomelo, grapefruit Tropical fruits: pineapple, watermelon, melon, fresh figs Berries: raspberries, strawberries, blueberries, currants Apples and pears Dried fruit, e.g. cranberries, apricots, figs, sultanas, prunes
Fruit preparations	Jams, compotes, preserves, plum jam
Cereal products	light bread, toasted bread, plain rolls, butter croissants Dark bread, wholemeal bread, rye bread, bread with grains Coarse groats, e.g. buckwheat, pearl barley, brown rice, whole-grain pasta Small-grain cereals, e.g. semolina, white rice, pasta, muesli, cornflakes, rice flakes
Animal fats	Butter, lard, bacon, mayonnaise
Vegetable fats	Olive oil Vegetable oils Vegetable margarine Cream, cream Dressings, salad dressings
Fast food and instant dishes	Burgers, nuggets, wraps, kebabs, pizza Chinese soups, instant noodles, jellies in a cup, sauces
Sweetened beverages	Fruit juices and nectars Vegetable and fruit juices, e.g. tomato, carrot and fruit juices Energy drinks Sweetened fizzy drinks, e.g. sprite, coca cola, pepsi, mirinda, fanta Coffee
Alcohol	Wine, vodka, beer, liqueurs

#### Statistical analysis

Statistical analysis was performed using Statistica 13.3 TIBCO software. Both parametric and non-parametric tests were used. The use of parametric tests was possible once their assumptions were met, i.e. the normality of the distributions of the variables under study, which was assessed with the Shapiro–Wilk W test. Comparisons between two groups for qualitative variables were made with the chi-square test, ordinal variables with the Mann–Whitney U test and quantitative variables with the Student's t-test for independent variables or alternatively with the non-parametric Mann–Whitney U test. Using the Spearman rank correlation coefficient, the correlation of two variables not meeting the normality criterion was calculated. A scale indicating the strength of the relationship was used to describe and interpret a significant correlation:

- r_xy_ = 0—no correlation;
- 0 < r_xy_ < 0.1—very weak correlation;
- 0.1 < r_xy_ < 0.3—weak correlation;
- 0.3 < r_xy_ < 0.5—average correlation;
- 0.5 < r_xy_ < 0.7—high correlation;
- 0.7 < r_xy_ < 0.9—very high correlation;
- 0.9 < r_xy_ < 1—correlation almost complete.

A value of p < 0.05 was taken as the level of statistical significance.

## Results

Most subjects were of normal weight (28 subjects—50.9%) or overweight (17 subjects—30.9%). There were no differences between the BMI of the subjects in the two groups (p = 0.210) (Table 3).

**Table 3:** Interpretation of BMI for both groups.

BMI	Study group		Control group		Total	
	N	%	N	%	N	%
Underweight	2	7.1	1	3.7	3	5.5
Standard	12	42.9	16	59.3	28	50.9
Overweight	8	28.6	9	33.3	17	30.9
Obesity	6	21.4	1	3.7	7	12.7
Total	28	100.0	27	100.0	55	100.0
p	χ^2^(3) = 4.51; p = 0.210					

N: number of observations; χ^2^: value of Pearson's chi-square test; p: test probability ratio; < 18.5: underweight; 18.5–24.9: standard; 25.0–29.9: overweight; > 30.0: obesity.

The following parameters were significantly higher in the test group: body fat (BF(%)) p = 0.004; metabolic age (MA) p = 0.004; pulse p = 0.001; body fat corpus (BFC (%)) p = 0.004; left arm fat (LAF(%)) p = 0.002; right arm fat (RAF(%)) p < 0.001; left leg fat (LLF(%)) p = 0.004; right leg fat (RLF(%)) p = 0.001. The control group had significantly higher values for the following parameters (p < 0.05): muscle mass (MM(kg)) p = 0.001; muscle quality (MQ(mq)) p = 0.001; body type (BT) p = 0.024; bone mass (BM(kg)) p = 0.005; primary metabolic rate (PMR) p = 0.010; body water content (BWC) p = 0.001; muscle mass right leg (MMRL(kg)) p = 0.007; muscle quality left leg (MQLL(mq)) p = 0.001; muscle quality right leg (MQRL (mq)) p = 0.001 (Table 4).

**Table 4:** Results of body mass composition measurements, detailing the two groups of subjects.

	Study group					Control group					t/Z*	p
	X	Me	Min	Max	S	X	Me	Min	Max	S		
BMI	25.9	25.1	15.4	36.8	5.2	24.2	23.0	17.6	33.2	3.3	1.47	0.146
BF (%)	28.7	28.1	8.2	48.1	10.2	21.6	20.2	12.0	40.0	6.6	3.02	**0.004**
MM (kg)	46.1	46.1	29.3	58.9	7.0	54.4	55.8	34.2	71.6	10.8	− 3.42	**0.001**
MQ (mq)	52.1	49.0	27.0	100.0	14.8	62.0	62.0	40.0	87.0	10.7	− 3.35*	**0.001**
BT	3.6	3.0	1.0	8.0	1.9	4.8	5.0	1.0	9.0	1.6	− 2.26*	**0.024**
BM (kg)	2.5	2.5	1.9	2.9	0.3	2.8	2.9	1.8	3.7	0.5	− 2.94	**0.005**
VF	5.8	5.3	1.0	20.0	3.9	4.3	4.0	1.0	13.0	3.1	1.72*	0.086
PMR	1507	1495	1141	1918	2042	1696	1706	1095	2354	314.8	− 2.66	**0.010**
MA	36.8	36.5	11.0	76.0	13.3	26.9	25.0	12.0	55.0	11.0	3.01	**0.004**
BWC (%)	52.2	51.0	41.1	65.5	6.3	57.9	58.1	48.7	68.3	5.5	− 3.57	**0.001**
Pulse	91.5	91.5	39.8	130.0	18.1	78.7	80.0	66.0	94.0	7.2	3.43	**0.001**
BFC (%)	27.9	27.2	8.8	51.4	9.9	21.0	21.5	10.8	37.4	6.2	3.05	**0.004**
LAF (%)	28.1	26.5	4.8	57.3	11.3	18.7	17.1	2.3	42.8	10.2	3.23	**0.002**
RAF (%)	30.2	29.7	5.7	49.6	11.1	18.7	16.6	1.9	41.7	8.8	4.25	** < 0.001**
LLF (%)	32.0	32.9	10.2	61.2	12.2	22.9	18.0	5.6	43.5	10.2	3.00	**0.004**
RLF (%)	32.6	35.1	10.2	51.4	11.5	22.2	18.8	5.3	43.5	10.5	3.46	**0.001**
MMC (kg)	26.4	25.6	19.3	41.1	4.9	30.0	30.3	19.5	41.5	5.4	*− 2.69	**0.007**
MMLA (kg)	2.6	2.6	1.7	4.9	0.7	3.0	3.1	1.6	4.5	0.9	*− 1.87	**0.062**
MMRA (kg)	2.5	2.5	1.8	4.3	0.5	2.9	3.1	1.6	4.8	0.8	*− 2.25	**0.025**
MMLL (kg)	7.6	7.6	2.2	10.2	1.5	9.0	9.4	5.7	13.4	2.0	*− 2.45	**0.014**
MMRL (kg)	7.7	7.7	5.8	9.3	1.0	9.2	9.8	5.9	12.8	2.0	*− 2.69	**0.007**
MQLA (mq)	53.6	51.5	12.0	100.0	20.8	61.4	60.0	42.0	88.0	12.1	− 1.69	0.096
MQRA (mq)	53.6	52.0	14.0	100.0	17.3	61.2	64.0	44.0	83.0	10.5	− 1.96	0.056
MQLL (mq)	50.6	48.5	29.0	89.0	12.3	62.8	59.0	38.0	87.0	12.7	− 3.62	**0.001**
MQRL (mq)	48.9	49.5	20.0	93.0	14.1	62.0	60.0	37.0	92.0	12.5	− 3.62	**0.001**

X: arithmetic mean; Me: median; Min: minimum; Max: Maximum; S: sample standard deviation; t-value of Student's t: test for independent variables; *Z: value of Mann–Whitney U-test; p: test probability ratio; BMI: body mass index; BF (%): body fat; MM (kg): muscle mass; MQ (mq): muscle quality; BT: body type; BM (kg): bone mass; VF: visceral fat; PMR: primary metabolic rate; MA: metabolic age; BWC (%): body water content; BFC (%): body fat corpus; LAF (%): left arm fat; RAF (%): right arm fat; LLF (%): left leg fat; RLF (%): right leg fat; MMC (kg): muscle mass corpus; MMLA (kg): muscle mass left arm; MMRA (kg): muscle mass right arm; MMLL (kg): muscle mass left leg; MMRL (kg): muscle mass right leg; MQLA (mq): muscle quality left arm; MQRA (mq): muscle quality right arm; MQLL (mq): muscle quality left leg; MQRL (mq): muscle quality right leg.

Significant values are in bold.

Subjects in the study group were significantly more likely to eat vegetables (p = 0.004) and animal fats (p = 0.027). In contrast, control subjects were more likely to eat fast-food and instant products (p = 0.006), drink sweetened beverages (p < 0.001) and alcohol (p < 0.001) (Table 5).

**Table 5:** Eating habits, detailing both groups of respondents.

	Study group					Control group					t/Z*	p
	X	Me	Min	Max	S	X	Me	Min	Max	S		
Sugar and sweets	3.0	3.0	1.4	4.6	0.8	2.8	2.6	2.0	4.0	0.6	1.04	0.297
Salty snacks	2.4	2.0	1.0	4.0	1.0	2.3	2.0	1.0	5.0	1.0	0.24	0.812
Milk and milk products	2.8	2.8	1.0	4.7	1.1	2.7	2.7	1.7	4.2	0.6	0.24	0.807
Eggs	2.8	3.0	1.0	5.0	1.2	2.9	2.0	1.0	5.0	1.2	− 0.24	0.806
Red meat and meat products	2.4	2.5	1.6	3.3	0.5	2.4	2.4	1.7	3.1	0.4	− 0.46	0.648
White meat and fish	2.9	3.0	1.3	4.0	0.7	2.6	2.3	1.7	3.7	0.6	1.88	0.061
Vegetables	3.2	3.4	1.7	4.2	0.5	2.9	2.9	2.0	3.8	0.5	**2.84**	**0.004**
Nuts and grains	2.5	3.0	1.0	4.0	1.0	2.5	2.5	1.0	4.0	0.9	0.34	0.733
Fruit	3.3	3.3	1.0	4.3	0.8	3.1	3.2	1.8	4.3	0.7	1.11	0.265
Fruit preparations and dried fruit	2.8	2.5	1.0	4.5	1.0	3.3	3.5	1.5	5.0	1.0	− 1.67	0.094
Cereal products	3.4	3.5	1.8	4.5	0.7	3.2	3.3	2.0	4.3	0.6	1.48	0.140
Animal fats	2.8	2.9	1.8	4.3	0.6	2.4	2.3	1.3	4.5	0.7	**2.21**	**0.027**
Vegetable fats	2.8	2.8	1.0	4.3	0.9	2.7	2.7	1.3	4.3	0.7	0.71	0.475
Fast-food and instant products	1.9	2.0	1.0	3.0	0.5	2.4	2.3	1.3	4.0	0.7	**− 2.76**	**0.006**
Sweetened beverages	2.5	2.6	1.0	5.0	0.8	3.4	3.5	1.0	4.8	0.9	**− 3.62**	** < 0.001**
Alcohol	1.0	1.0	1.0	1.0	0.0	2.2	2.0	1.0	4.0	1.0	**− 5.12**	** < 0.001**

X: arithmetic mean; Me: median; Min: minimum; Max: maximum; S: sample standard deviation; t: value of Student's t-test for independent variables; *Z: value of Mann–Whitney U-test; p: test probability ratio.

Significant values are in bold.

Only those in the control group supplemented protein and creatine (p < 0.001 and p = 0.001). In contrast, those in the control group drank more water per day (p = 0.007). All respondents in the control group and an average of one in three in the study group prepared their own meals. This difference was statistically significant (p < 0.001) (Table 6).

**Table 6:** Characteristics of supplementation, amount of water drunk and method of meal preparation, with details of both groups.

	Study group		Control group		P	
	N	%	N	%		
Magnesium	10	35.7	9	33.3	χ^2^(1) = 0.03 p = 0.852	
Potassium	2	7.1	5	18.5	χ^2^(1) = 1.60 p = 0.205	
Protein	0	0.0	15	55.6	**χ**^**2**^**(1) = 21.38 p < 0.001**	
Vitamin D	18	64.3	16	59.3	χ^2^(1) = 0.15 p = 0.701	
Vitamin B12	6	21.4	8	29.6	χ^2^(1) = 0.48 p = 0.485	
Vitamin C	11	39.3	8	29.6	χ^2^(1) = 0.57 p = 0.451	
Iron	2	7.1	6	22.2	χ^2^(1) = 2.51 p = 0.112	
Omega acids	12	42.9	9	33.3	χ^2^(1) = 0.52 p = 0.467	
Creatine	0	0.0	9	33.3	**χ**^**2**^**(1) = 11.15 p = 0.001**	
Zinc	2	7.1	5	18.5	χ^2^(1) = 1.60 p = 0.205	

The number of litres of water consumed per day	Study group		Control group		Total	
	N	%	N	%	N	%
0.5–1 L	8	28.6	1	3.7	9	16.4
1–2 L	9	32.1	11	40.7	20	36.4
2–3 L	9	32.1	5	18.5	14	25.5
> 3 L	2	7.1	10	37.0	12	21.8
Total	28	100.0	27	100.0	55	100.0
p	χ^2^(3) = 12.11 p = 0.007					

How to prepare meals for oneself	Study group		Control group		Total	
	N	%	N	%	N	%
By oneself	9	32.1%	27	100.0%	36	65.5%
With the help of relatives	19	67.9%	0	0.0%	19	34.6%
Total	28	100.0%	27	100.0%	55	100.0%
p	χ^2^(1) = 27.99 p < 0.001					

N: number of observations; χ^2^: value of Pearson's chi-square test; p: test probability ratio.

Significant values are in bold.

Using Spearman's rank correlation coefficient, the relationship between body composition scores and dietary habits in the study group was examined. Significant negative correlations with a medium strength of association were found, meaning: the less frequent the consumption of fast-food and instant products (14), the lower the percentage of body fat (BF (%)) (R = − 0.4) and the amount of right leg fat (RLF (%)) (R = − 0.4); less frequent consumption of processed fruit and dried fruit (10), the lower the muscle mass (MM (kg)) (R = − 0.4) and the amount of muscle mass corpus (MMC (kg)), and the lower the rate of primary metabolic rate (PMR) (R = − 0.4); less frequent consumption of red meat and meat products (5), the lower the body type number (BT), indicating overweight or obesity (R = − 0.4); less frequent consumption of animal fats (12), the lower the bone mass (BM (kg)) (R = − 0.4); less frequent consumption of dairy products, the lower the metabolic age (MA) (R = − 0.4).

Positive correlations with average strength of association were also found, meaning: the higher frequent the consumption of fast-food and instant products (14), the higher the percentage of body water content (BWC) (R = 0.4); the higher frequent the consumption of red meat and meat products (5) and processed fruit and dried fruit (10), the higher the percentage of body fat (BF (%)) in the left arm (LAF (%)) (R = 0.4) and (R = 0.5) and in the right arm (RLF (%)) (R = 0.4) and (R = 0.6), the percentage of body fat in the left leg (LLF (%)) and the consumption of fruit and dried fruit preparations (10) (R = 0.4).

Further positive mean correlations were noted between sugar and sweet consumption (1) and poorer muscle quality in the left arm (MQLA (mq)) (R = 0.4), as well as poorer muscle quality in the right arm (MQRA (mq)) (R = 0.4), salty snacks (2) and poorer muscle quality in the right leg (MQRL (mq)) (R = 0.4) (Table 7).

**Table 7:** Evaluation of the relationship between body mass composition scores and dietary habits in the study group.

*R	1	2	3	4	5	6	7	8	9	10	11	12	13	14	15
BMI	0.4	0.2	− 0.2	0.1	− 0.2	0.2	0.0	0.1	0.0	− 0.1	− 0.2	− 0.2	0.3	0.1	− 0.1
BF (%)	0.1	− 0.2	− 0.2	0.1	− 0.1	0.3	0.0	− 0.2	− 0.1	0.3	− 0.1	0.1	− 0.3	**− 0.4**	− 0.2
MM (kg)	0.2	0.1	0.0	− 0.3	− 0.3	− 0.1	0.1	0.1	− 0.2	**− 0.4**	0.0	− 0.3	0.2	0.3	0.1
MQ (mq)	0.1	0.2	− 0.1	0.1	0.2	0.0	− 0.2	0.2	0.1	− 0.3	− 0.3	− 0.2	0.3	0.3	0.0
BT	− 0.3	− 0.1	0.1	− 0.4	**− 0.4**	0.0	0.2	0.1	− 0.1	0.0	0.0	− 0.2	0.1	0.0	0.0
BM (kg)	0.1	0.1	− 0.1	− 0.3	− 0.3	− 0.2	0.2	0.1	− 0.1	− 0.3	0.1	**− 0.4**	0.2	0.3	0.2
VF	0.3	0.2	− 0.2	− 0.1	− 0.2	0.0	0.2	− 0.1	0.0	0.0	0.0	− 0.3	0.4	− 0.2	− 0.3
PMR	0.2	0.1	0.0	− 0.3	− 0.3	− 0.1	0.1	0.1	− 0.2	**− 0.4**	0.0	− 0.3	0.1	0.3	0.2
MA	0.3	0.1	**− 0.4**	0.1	− 0.1	0.0	0.0	− 0.1	− 0.1	0.2	− 0.1	− 0.2	0.3	− 0.2	− 0.2
BWC (%)	0.3	0.0	0.2	− 0.1	0.0	0.1	0.1	0.1	0.1	− 0.3	0.2	0.0	0.2	**0.4**	0.1
Pulse	0.0	− 0.2	0.0	0.1	− 0.3	0.3	− 0.2	0.0	− 0.2	0.0	0.3	0.0	0.1	− 0.1	− 0.2
BFC (%)	0.3	0.0	− 0.3	0.2	0.0	0.2	0.1	− 0.2	− 0.1	0.3	− 0.2	0.0	0.0	− 0.2	− 0.2
LAF (%)	− 0.1	0.2	− 0.2	0.4	**0.4**	0.0	− 0.1	− 0.2	0.1	**0.5**	− 0.1	0.3	− 0.2	− 0.2	− 0.2
RAF (%)	− 0.1	0.2	− 0.2	0.3	**0.4**	− 0.1	0.0	− 0.2	0.1	**0.6**	− 0.1	0.3	− 0.2	− 0.2	− 0.2
LLF (%)	− 0.2	0.0	− 0.2	0.3	0.2	0.0	− 0.2	− 0.2	0.2	**0.4**	− 0.1	0.1	− 0.3	− 0.3	− 0.1
RLF (%)	− 0.2	0.1	− 0.1	0.3	0.1	0.1	− 0.2	− 0.2	0.1	0.4	− 0.1	0.1	− 0.2	**− 0.4**	− 0.2
MMC (kg)	0.2	0.1	0.0	− 0.3	− 0.3	− 0.1	0.1	0.1	− 0.2	**− 0.4**	0.0	− 0.4	0.1	0.3	0.2
MMLA (kg)	0.2	0.1	− 0.1	− 0.3	− 0.4	− 0.1	0.2	0.2	− 0.1	− 0.3	0.1	− 0.3	0.3	0.2	0.1
MMRA (kg)	0.2	0.1	− 0.1	− 0.3	− 0.4	− 0.1	0.2	0.2	− 0.1	− 0.3	0.1	− 0.3	0.3	0.2	0.1
MMLL (kg)	0.2	0.2	− 0.1	− 0.3	− 0.2	− 0.1	0.2	0.2	− 0.1	− 0.3	0.1	− 0.2	0.3	0.3	0.1
MMRL (kg)	0.2	0.1	0.0	− 0.3	− 0.3	− 0.1	0.2	0.2	− 0.1	− 0.3	0.0	− 0.2	0.2	0.3	0.1
MQLA (mq)	**0.4**	− 0.2	− 0.3	− 0.1	0.0	0.1	0.1	0.0	0.0	− 0.1	− 0.3	− 0.2	0.1	0.0	0.0
MQRA (mq)	**0.4**	− 0.1	− 0.3	0.1	0.0	0.1	0.0	0.0	0.1	− 0.1	− 0.2	− 0.2	0.2	0.0	0.0
MQLL (mq)	− 0.2	0.4	0.1	0.2	0.2	− 0.1	− 0.3	0.2	0.2	− 0.2	− 0.2	0.0	0.4	0.3	− 0.1
MQRL (mq)	− 0.2	**0.4**	0.1	0.2	0.4	− 0.1	− 0.3	0.2	0.2	− 0.1	− 0.2	0.1	0.3	0.3	− 0.1

*R-value of Spearman's rank correlation/bold indicates statistically significant relationships at p < 0.05; 1: sugar and confectionery; 2: salt snacks; 3: milk and dairy products; 4: egg; 5: red meat and meat products; 6: white meat and fish; 7: vegetables; 8: nuts and grains; 9: fruits; 10: fruit preparations and dried fruit; 11: cereal products; 12: animal fats; 13: vegetable fats; 14: fast-food and instant foods; 15: sweetened beverages; BMI: body mass index; BF (%): body fat; MM (kg): muscle mass; MQ (mq): muscle quality; BT: body type; BM (kg): bone mass; VF: visceral fat; PMR: primary metabolic rate; MA: metabolic age; BWC (%): body water content; BFC (%): body fat corpus; LAF (%): left arm fat; RAF (%): right arm fat; LLF (%): left leg fat; RLF (%): right leg fat; MMC (kg): muscle mass corpus; MMLA (kg): muscle mass left arm; MMRA (kg): muscle mass right arm; MMLL (kg): muscle mass left leg; MMRL (kg): muscle mass right leg; MQLA (mq): muscle quality left arm; MQRA (mq): muscle quality right arm; MQLL (mq): muscle quality left leg; MQRL (mq): muscle quality right leg.

Using Spearman's rank correlation coefficient, the relationship between body composition scores and eating habits in the control group was examined. Significant negative correlations with a moderate strength of association were found, meaning that the less frequent the consumption of eggs (4), the lower the BMI (R = − 0.5) and the percentage of body fat (BF(%)) (R = − 0.5) and visceral fat (VF), (R = − 0.4). In addition, the less frequent the consumption of animal fats (12), the lower the visceral fat (VF) (R = − 0.4); the less frequent the consumption of sugar and sweets (1), the lower the basal metabolic rate (PMR) (R = − 0.4). In contrast, there was a positive association between primary metabolic rate (PMR) and fruit consumption (9) (R = 0.5); the higher the PMR, the higher the fruit consumption (9); the lower the metabolic age (MA), the less frequent the egg consumption (4) (R = − 0.4); the lower the percentage of body water (BWC (%)), the less frequent the consumption of milk and dairy products (3) (R = − 0.4); the lower the percentage of body fat (BF (%)), the less frequent the consumption of eggs (4) (R = − 0.5); the lower the amount of muscle mass in the left arm (MMLA (kg)) the less frequent the consumption of animal fats (12) (R = − 0.4). In contrast, the higher the frequency of fruit consumption (9), the higher the amount of muscle mass in the left leg (MMLL (kg)) (R = 0.4) and muscle mass in the right leg (MMRL (kg)) (R = 0.4) (Table 8).

**Table 8:** Evaluation of the relationship between body mass composition scores and eating habits in the control group.

*R	1	2	3	4	5	6	7	8	9	10	11	12	13	14	15
BMI	− 0.1	− 0.2	− 0.1	**− 0.5**	0.2	0.1	− 0.1	0.1	− 0.1	0.2	0.1	− 0.2	− 0.2	− 0.1	− 0.1
BF (%)	0.0	− 0.3	0.1	**− 0.5**	0.3	− 0.1	0.0	0.1	− 0.1	0.1	0.0	− 0.2	− 0.2	0.0	− 0.1
MM (kg)	− 0.3	− 0.1	− 0.1	0.0	0.1	0.1	0.0	0.2	0.3	0.2	0.2	− 0.2	0.1	0.2	− 0.2
MQ (mq)	0.0	− 0.3	0.0	0.1	0.2	− 0.1	0.0	− 0.2	− 0.1	− 0.1	0.0	0.1	− 0.3	0.0	− 0.1
BT	0.3	0.1	0.2	0.1	0.0	0.0	0.1	0.1	0.0	0.3	0.1	0.4	0.0	− 0.3	0.3
BM (kg)	**− 0.4**	− 0.1	− 0.1	− 0.1	0.0	0.2	− 0.1	0.3	0.3	0.2	0.3	− 0.3	0.0	0.0	− 0.2
VF	− 0.2	− 0.4	− 0.2	**− 0.4**	0.0	0.1	− 0.2	0.2	0.0	0.1	0.2	**− 0.4**	− 0.1	0.0	− 0.2
PMR	**− 0.4**	− 0.1	0.1	0.0	0.1	0.2	− 0.1	0.3	**0.5**	0.2	0.3	− 0.2	0.0	0.0	− 0.3
MA	− 0.1	− 0.1	− 0.1	**− 0.4**	0.1	0.0	− 0.2	0.1	− 0.1	0.0	0.0	− 0.2	− 0.2	0.1	− 0.3
BWC (%)	− 0.1	− 0.1	**− 0.4**	0.1	− 0.3	− 0.2	− 0.1	0.0	− 0.1	− 0.1	− 0.1	0.0	0.2	0.2	0.1
Pulse	0.2	0.0	− 0.1	− 0.2	0.2	− 0.1	0.2	0.2	− 0.2	− 0.2	− 0.3	0.0	0.1	0.0	0.2
BFC (%)	0.0	− 0.2	0.1	**− 0.5**	0.2	0.0	− 0.1	0.2	0.1	0.1	0.0	− 0.3	− 0.2	0.2	− 0.1
LAF (%)	− 0.1	− 0.1	0.3	− 0.1	0.2	0.1	0.2	− 0.1	− 0.1	0.1	0.0	− 0.1	0.0	− 0.1	0.1
RAF (%)	0.0	− 0.1	0.1	− 0.2	0.2	0.1	0.1	0.1	0.0	0.1	0.0	− 0.2	− 0.2	− 0.1	0.0
LLF (%)	0.1	− 0.3	0.3	− 0.2	0.3	0.0	0.3	0.0	− 0.2	0.1	0.1	0.0	− 0.1	− 0.1	0.1
RLF (%)	0.0	− 0.2	0.2	− 0.2	0.3	0.1	0.2	0.1	− 0.1	0.1	0.1	− 0.2	− 0.1	− 0.1	0.0
MMC (kg)	− 0.2	− 0.1	0.0	0.0	− 0.2	0.1	− 0.1	0.0	0.2	0.1	0.2	− 0.2	0.0	0.1	− 0.3
MMLA (kg)	− 0.2	− 0.1	− 0.3	− 0.3	0.0	0.1	− 0.2	0.1	0.1	0.0	0.1	**− 0.4**	− 0.2	0.1	− 0.1
MMRA (kg)	− 0.2	0.1	− 0.1	− 0.2	0.0	0.3	− 0.1	0.1	0.1	0.0	0.2	− 0.3	0.0	0.0	0.0
MMLL (kg)	− 0.3	− 0.1	− 0.1	− 0.2	0.0	0.3	− 0.1	0.3	**0.4**	0.1	0.2	− 0.4	0.0	0.1	− 0.1
MMRL (kg)	− 0.3	− 0.1	− 0.1	− 0.1	0.0	0.2	− 0.2	0.3	**0.4**	0.1	0.1	− 0.2	− 0.1	0.0	− 0.2
MQLA (mq)	0.2	0.1	− 0.1	− 0.1	0.1	− 0.2	0.0	− 0.1	− 0.1	− 0.1	− 0.4	0.2	− 0.4	0.1	− 0.2
MQRA (mq)	0.3	− 0.1	0.4	− 0.1	0.3	0.0	0.0	− 0.2	0.1	0.2	0.1	0.2	− 0.1	0.1	− 0.1
MQLL (mq)	− 0.1	− 0.1	0.1	0.0	0.1	0.0	0.1	− 0.4	− 0.3	− 0.1	0.2	− 0.1	0.0	− 0.1	0.0
MQRL (mq)	0.0	− 0.1	0.0	0.2	0.0	0.1	0.1	− 0.3	− 0.3	0.0	0.2	0.1	0.0	− 0.2	0.0

*R-value of Spearman's rank correlation/bold indicates statistically significant relationships at p < 0.05; 1: sugar and confectionery; 2: salt snacks; 3: milk and dairy products; 4: egg; 5: red meat and meat products; 6: white meat and fish; 7: vegetables; 8: nuts and grains; 9: fruits; 10: fruit preparations and dried fruit; 11: cereal products; 12: animal fats; 13: vegetable fats; 14: fast-food and instant foods; 15: sweetened beverages; BMI: body mass index; BF (%): body fat; MM (kg): muscle mass; MQ (mq): muscle quality; BT: body type; BM (kg): bone mass; VF: visceral fat; PMR: primary metabolic rate; MA: metabolic age; BWC (%): body water content; BFC (%): body fat corpus; LAF (%): left arm fat; RAF (%): right arm fat; LLF (%): left leg fat; RLF (%): right leg fat; MMC (kg): muscle mass corpus; MMLA (kg): muscle mass left arm; MMRA (kg): muscle mass right arm; MMLL (kg): muscle mass left leg; MMRL (kg): muscle mass right leg; MQLA (mq): muscle quality left arm; MQRA (mq): muscle quality right arm; MQLL (mq): muscle quality left leg; MQRL (mq): muscle quality right leg.

## Discussion

Eating habits have a direct impact on body composition. The study and control groups had different dietary patterns, resulting in differences in parameters such as body fat percentage, muscle mass and bone mass. Correlation analysis in the study group showed negative correlations between body composition parameters and some dietary habits. Less frequent consumption of fast food and processed fruits correlated with lower body fat percentage and metabolic rate. In contrast, positive correlations were observed between higher consumption of fast food, instant products and red meat, poorer muscle quality and higher body fat percentage. Similarly, in the control group, negative correlations were found between body composition parameters and certain eating habits, such as less frequent consumption of eggs and lower BMI and body fat percentage. In contrast, positive associations were found between higher fruit consumption and basal metabolism. The topic of the relationship between body composition and the eating habits of physically active people with disabilities is extremely important and timely. For people with disabilities, body composition can affect mobility, stability, muscle strength and overall physical fitness.

Body composition analysis is crucial in assessing physiological and pathological states in populations due to its importance as a determinant of health states and nutritional indicators^14^. A review of studies by Hassan et al. confirms the positive effects of diet and physical activity on the physical fitness of people with disabilities^15^. Adequate muscle mass can support musculoskeletal function and help counteract potential health problems^16^. The study Sawada et al. found that reduced amounts of these muscles were associated with poorer basic activities of daily living (ADL) and also a depressive state^17^. Among individuals with physical or cognitive disabilities, physical activity showed a positive association with cardiovascular and respiratory fitness, muscle strength, functional capabilities, psychosocial well-being, and markers of cardiometabolic health^18^. The WHO guidelines on physical activity and sedentary behavior provide evidence that physical activity tailored to the type of disability, along with guidelines limiting sedentary behavior, contribute to improved health and functioning among individuals with disabilities, similar to the benefits experienced by able-bodied individuals^19^. It is worth mentioning that people who have been physically active throughout their adult lives are less likely to suffer from physical disability in old age compared to their less active peers^18^. A study by Inukai et al. suggests that exercise is effective in reducing waist circumference, waist-to-hip ratio and body fat percentage in people after spinal cord injury, and such effects may help improve sports performance and possibly protect against the development of metabolic syndromes resulting from a sedentary lifestyle^20^. A study by Cavedon et al. found that regular practice of basketball by women with disabilities can help reduce fat accumulation associated with physical disability^21^. In contrast, a study by Medeiros et al. on disabled swimmers showed that after a period of six months of training, a reduction in fat mass and an increase in lean body mass were observed, which translated into swimming performance^22^.

A study among people with intellectual disabilities showed that the prevalence of overweight and obesity was similar in men and women^14^. In our study, there were no differences between the BMI of the subjects in the two groups. In contrast, in a study by Skrzypek et al. on children and adolescents with intellectual disabilities, high body weight was found in 66.7%, including overweight in 27.8% and obesity in 38.9%^23^. In the Bede et al. study, the number of underweight subjects, i.e. 5%, is similar to our results, the percentage of overweight subjects is 30.9%, while the number of obese subjects is 12.7%^24^. In contrast, in our study, one-third of the subjects were overweight and exactly the same percentage of subjects were obese as in the aforementioned study.

A study by Riddle et al. showed that the inability to prepare meals independently was associated with disability^25^. As also shown in our own study, where two-thirds of the study group prepared meals with the help of relatives. Research^7^ shows that as many as 9% of Poles do not eat or eat fresh fruit very rarely, and only 21% of Poles eat fresh fruit regularly. Based on the results of our research, we see that fruit is eaten on average several times a month. In contrast, a study assessing the frequency of consumption of vegetables, fruit and fast food among adolescents found that as many as 34.5% of respondents ate fruit less than once a day^26^. Turning our attention to vegetable consumption, we also note that only 30% of Poles consume fresh vegetables once a day, which is associated with deficiencies in macro- and microelements, which in turn may contribute to the development of civilisation diseases^7^. Our research shows that vegetable consumption is at a very low level, as vegetables are consumed on average several times a month. In contrast, in a study by Beal et al. vegetables are consumed less than once a day by one-fifth of the respondents^26^. Sweets are consumed once a day by one-third of Poles, while sweets are consumed once on several days by 37% of Poles, which indicates that the level of sweets consumption among Poles is not high^7^. Our research shows that people with disabilities reach for sweets more often and this is on average several times a month, while people in the control group consume sweets less than several times a month. Fast-food products are consumed once a week by as many as 46.1%^26^, and from the results of our study we learn that people with disabilities reach for fast food never or almost never, while people without disabilities do so more often. The results of Bede et al.'s study, which focused on assessing the dietary practices of students, show that half of the respondents consume only two meals a day^24^, while our study shows that two meals a day are consumed by 12.7% of the respondents, which is a significant difference. Snacks are consumed daily by 40.8% of the students, while meat is consumed by 21.3% of the respondents^26^, our research shows that snacks and red meat are consumed less often than a few times a month, the respondents are more likely to go for white meat and fish. A study by Garrido-Miguel et al. in 355 students between the ages of 18 and 30 showed that egg consumption has a significant effect on body composition, and that this is mainly due to the protein content of eggs^27^. Our study also found a positive association between egg consumption and body composition of the subjects. Lower egg consumption may be associated with lower BMI, lower body fat, lower visceral fat and lower metabolic age. Khodayari's study showed that consumption of poultry and white meat has an impact on overall obesity, while consumption of processed meat has an impact on central obesity^28^. Our study showed that those who consumed red meat and processed meat had higher left and right arm body fat, our study confirms the effect of processed meat consumption on the development of central obesity.

Mitra et al. proved that more than 3 L of water is consumed by as many as 53.6% of the subjects^29^, while our study showed that 3 L of water per day is consumed by more than twice as many subjects, i.e. only 21.8%. Porro et al. investigating coffee consumption, showed that 22.1% of those surveyed did not regularly consume coffee, while 58.4% consumed between 0.5 and 3 cups of coffee per day and 19.5% consumed more than 3 cups per day^30^. Our research indicates that 21.8% of respondents consume coffee once a day, 16.4% consume coffee twice a day, 9.1% consume coffee three times a day and 52.8% of respondents drink coffee more than three times a day. The studies are in line.

A study analysing the nutritional status of young people with disabilities showed that all subjects had an excessive intake of vitamins B1, B2, B3, B6, B12, A and vitamin C in the majority of subjects, all subjects were deficient in vitamin D. Excessive intakes of sodium, phosphorus and magnesium were also found, while calcium and iodine were deficient^23^. Our study showed that vitamin B12 is supplemented by more than one-fifth of the subjects in the study group and almost one-third of those in the control group. Vitamin D, on the other hand, is taken by 64.3% of the study group and more than half of the control group. Protein is supplemented by more than half of the control group; no one in the study group supplements protein or creatine. Vitamin C is supplemented by 19 people in the study and control group combined. The study by de la Puente Yagüe et al. confirmed the positive effect of vitamin D supplementation on muscle recovery after exercise. An increase in muscle strength and a reduction in the occurrence of injuries and trauma were noted in the subjects^31^.

## Summary

The study investigated the relationship between body composition metrics and dietary habits in both a study group and a control group. Several significant findings emerged, shedding light on how dietary choices relate to various aspects of body composition and metabolism. In the study group, individuals exhibited higher body fat percentages, metabolic age, pulse rates, and fat distribution across limbs compared to the control group. Notably, their dietary habits leaned towards vegetable and animal fat consumption. Conversely, the control group showed higher values in parameters related to muscle mass, muscle quality, bone mass, primary metabolic rate, and body water content. Their dietary habits were characterized by a preference for fast-food, instant products, sweetened beverages, and alcohol. Additionally, the control group supplemented their diet with protein and creatine, while also consuming more water daily and showing a higher tendency to prepare their own meals. The body composition of people with disabilities differs significantly from their non-disabled counterparts, often showing a higher percentage of body fat. For people with disabilities, eating processed fruits, dried fruits, fast food and red meat is not recommended due to the possibility of higher body fat. On the other hand, in non-disabled people, he does not recommend frequent consumption of eggs, animal fats and sugar and sweets due to the possibility of higher body fat, visceral fat and higher BMI.

Overall, the findings underscore the significant impact of dietary habits on body composition and metabolism. They highlight the importance of balanced dietary choices, with an emphasis on whole foods and moderation in processed and high-fat options, for maintaining optimal body composition and metabolic health. These insights could inform personalized dietary interventions aimed at improving overall health outcomes.

## Conclusion

1. Disabled people have a percentage of higher body fat and lower muscle mass compared to healthy people, even though the latter have a lower quality diet.
2. Consumption of fast food and instant and dried fruits, as well as sugars and sweets and salty snacks is unhealthy for people with disabilities, as it is associated with increased body fat and reduced muscle mass, and promotes poorer muscle quality.
3. In non-disabled people, consumption of sugar and sweets promotes lower bone mass, while lower egg consumption results in a lower BMI and a lower percentage of body fat, trunk fat, visceral fat and lowers metabolic age.
4. The consumption of animal fats and eggs should be recommended for people with low bone and muscle mass and those with a higher BMI and body fat and their consumption lowers these parameters.

## Data Availability

The datasets used and/or analysed during the current study available from the corresponding author on reasonable request.
